# Antibacterial activity of silver and zinc nanoparticles against *Vibrio cholerae* and enterotoxic *Escherichia coli*

**DOI:** 10.1016/j.ijmm.2014.11.005

**Published:** 2015-01

**Authors:** Wesam Salem, Deborah R. Leitner, Franz G. Zingl, Gebhart Schratter, Ruth Prassl, Walter Goessler, Joachim Reidl, Stefan Schild

**Affiliations:** a University of Graz, Institute of Molecular Biosciences, BioTechMed-Graz, Humboldtstrasse 50, A-8010 Graz, Austria; b South Valley University, Faculty of Science, Qena, Egypt; c Institute of Biophysics, Medical University of Graz, BioTechMed-Graz, Schmiedlstraße 6, 8042 Graz, Austria; d Institute for Chemistry, Analytical Chemistry, University of Graz, BioTechMed-Graz, 8010 Graz, Austria

**Keywords:** *Caltropis procera*, Nanoparticles, In vivo, Colonization, Infant mouse model, Biofilm, Minimal inhibitory concentration, Survival curve, Therapeutic agent, Antimicrobial activity

## Abstract

*Vibrio cholerae* and enterotoxic *Escherichia coli* (ETEC) remain two dominant bacterial causes of severe secretory diarrhea and still a significant cause of death, especially in developing countries. In order to investigate new effective and inexpensive therapeutic approaches, we analyzed nanoparticles synthesized by a green approach using corresponding salt (silver or zinc nitrate) with aqueous extract of *Caltropis procera* fruit or leaves. We characterized the quantity and quality of nanoparticles by UV–visible wavelength scans and nanoparticle tracking analysis. Nanoparticles could be synthesized in reproducible yields of approximately 10^8^ particles/ml with mode particles sizes of approx. 90–100 nm. Antibacterial activity against two pathogens was assessed by minimal inhibitory concentration assays and survival curves. Both pathogens exhibited similar resistance profiles with minimal inhibitory concentrations ranging between 5 × 10^5^ and 10^7^ particles/ml. Interestingly, zinc nanoparticles showed a slightly higher efficacy, but sublethal concentrations caused adverse effects and resulted in increased biofilm formation of *V. cholerae*. Using the expression levels of the outer membrane porin OmpT as an indicator for cAMP levels, our results suggest that zinc nanoparticles inhibit adenylyl cyclase activity. This consequently deceases the levels of this second messenger, which is a known inhibitor of biofilm formation. Finally, we demonstrated that a single oral administration of silver nanoparticles to infant mice colonized with *V. cholerae* or ETEC significantly reduces the colonization rates of the pathogens by 75- or 100-fold, respectively.

## Introduction

Recently, nanotechnology has become increasingly important in the biomedical and pharmaceutical areas as alternative antimicrobial strategy due to re-emergence infectious diseases and the appearance of antibiotic-resistant strains especially within Gram-negative microorganisms (Desselberger, 2000). Biosynthesis of green nanoparticles using plant extracts is an interesting area in the field of nanotechnology, which has economic and eco-friendly benefits over chemical and physical methods of synthesis (Suzan et al., 2014). Nanoparticles (NPs) are typically no greater than 100 nm in size and their biocidal effectiveness is suggested to be owing to a combination of their small size and high surface-to-volume ratio, which enable intimate interactions with microbial membranes (Allaker, 2010; Morones et al., 2005). In addition, inorganic antibacterial agents such as metal and metal oxides are advantageous compared to organic compound due to their stability (Sawai, 2003; Sondi and Sondi, 2004). Among these metal oxides, ZnO has attracted a special attention as antibacterial agent. For instance, ZnO inhibits the adhesion and internalization of enterotoxigenic *E. coli* (ETEC) into enterocytes (Roselli et al., 2003). In addition, ZnO nanoparticles (ZnO-NPs) exhibit antibacterial activity and can reduce the attachment and viability of microbes on biomedical surfaces (Brayner et al., 2006; Yamamoto, 2001). Interestingly, several results suggest a selective toxicity of ZnO-NPs preferentially targeting prokaryotic systems, although killing of cancer cells has also been demonstrated (Hanley et al., 2008; Reddy et al., 2007; Taccola et al., 2011). Several mechanisms have been reported for the antibacterial activity of ZnO-NPs. For example ZnO-NPs can interact with membrane lipids and disorganize the membrane structure, which leads to loss of membrane integrity, malfunction, and finally to bacterial death (Krishnamoorthy et al., 2012; Zhang et al., 2007). ZnO may also penetrate into bacterial cells at a nanoscale level and result in the production of toxic oxygen radicals, which damage DNA, cell membranes or cell proteins, and may finally lead to the inhibition of bacterial growth and eventually to bacterial death (Apperlot et al., 2009; Irzh et al., 2010; Makhulf et al., 2005; Moody and Hassan, 1982; Zhang et al., 2007).

Furthermore, Ag^+^ ions and Ag-based compounds are highly toxic to several microorganisms, which make them interesting candidates for multiple applications in the medical field (Furno et al., 2004; Prakash et al., 2013). Ag is generally used as nitrate salt, but in the form of Ag nanoparticles (Ag-NPs) the surface area is increased and thereby antimicrobial efficacy is greatly enhanced. Though Ag-NPs find use in many antibacterial applications, the action of this metal on microbes is not fully known. It has been hypothesized that silver nanoparticles can cause cell lysis or growth inhibition via various mechanisms (Kim et al., 2007; Prabhu and Poulose, 2012). The lethality of silver for bacteria can also be in part explained by thiol-group reactions that inactivate enzymes (Chen and Schluesener, 2008; Feng et al., 2000). Also, Steuber and colleagues suggested a mechanism for Ag^+^ action in *Vibrio alginolyticus* involving the direct displacement of FAD from the holo-enzyme Na^+^-NQR, which results in loss of enzyme activity (Steuber et al., 1997). In summary, silver treatment inhibits DNA replication, expression of ribosomal and other cellular proteins, and interferes with the bacterial electron transport chain (Bragg and Rainnie, 1974; Feng et al., 2000; Yamanaka et al., 2005).

Several reports demonstrated the synthesis of ZnO- and Ag-NPs from natural sources like plants or microorganisms by green chemistry approaches (Babu and Prabu, 2011). The use of plant extracts for nanoparticles synthesis may be advantageous over other biological processes, because it drops the elaborate process of maintaining cell cultures and can also be used for large-scale NPs synthesis (Jeeva et al., 2014). Additionally, the green chemistry approach for the synthesis of NPs using plants avoids the generation of toxic byproducts. Among the various known synthesis methods, plant mediated NPs synthesis is preferred as it is cost-effective, eco-friendly and safe for human therapeutic use (Kumar and Yadav, 2009).

Diarrheal diseases are still a common worldwide cause of morbidity and mortality especially in the developing world. Within these areas, *V. cholerae* (∼25%) followed by ETEC (∼15%) are most prevalent bacterial pathogens causing diarrheal diseases (Chowdhury et al., 2011; Walker et al., 2007). *V. cholerae* is the causative agent of cholera, a life-threatening secretory diarrheal disease. According to Southeastern and Central Asia reports the annual acute diarrheal cases for *V. cholerae* infection were estimated more than 1 million (WHO, 2013). ETEC is a common cause of traveler's diarrhea, being responsible for up to one-half of diarrheal episodes in travelers to Asia, Africa and Latin America (Gupta et al., 2008; Qadri et al., 2005; Sanchez and Holmgren, 2005; Tobias et al., 2011). Particularly children show a high mortality rate in developing countries where diarrheal diseases remain the second most common cause of death (Levine, 2006). Even today treatment of these diarrheal diseases relies on a simple rehydration therapy, sometimes in combination with antimicrobial agents (Sack et al., 2004). The rehydration therapy is highly effective, but appropriate sterile solutions, antibiotics and medical expertise are not always available and during the explosive outbreaks medical facilities cannot cope with the massive numbers of incoming patients. Thus, alternative strategies should be investigated.

*Calotropis procera* is a shrub (F: Asclepiadaceae) distributed in West Africa, Asia and other parts of the tropics. The plant is erect, tall, large, branched and perennial with milky latex throughout (Irvine, 1961). Interestingly, Babu and Prabu recently described the Ag-NPs synthesis using aqueous extract of *Calotropis procera* flower, while the reduction was considered to occur due to the phenolics, terpenoids, polysaccharides and flavonoids present in the extract (Babu and Prabu, 2011).

In the present study, we synthesized metallic ZnO- and Ag-NPs using leaf and fruit extract of *Calotropis procera* and characterized their antibacterial activity against *V. cholerae* and ETEC. Especially Ag-NPs synthesized from leaf extracts showed the most robust antibacterial efficacy against both pathogens throughout the study. Furthermore, these Ag-NPs reduced fitness of the bacteria in biofilms as well as in vivo.

## Materials and methods

Bacterial strains, culture conditions and supplements. *V. cholerae* AC53 and ETEC H10407, spontaneous streptomycin-resistant (Sm^R^) derivatives of the clinical isolates O1 El Tor Ogawa E7946 (Miller et al., 1989); (Schild et al., 2007) or ETEC O78:H11:K80 (Evans and Evans, 1973), were used in this study. Unless stated otherwise strains were grown in LB broth with aeration at 37 °C or for biofilm formation under static conditions at room temperature (RT). If required, streptomycin was used with a final concentration of 100 μg/ml.

### Plant materials and preparation of the extracts

Healthy leaves and fruits of *Caltropis procera* were collected from South Valley University campus at Qena city (Egypt), washed thoroughly with tap water followed by distilled water, and air dried on a paper towel for 4–6 days. Dry leaves were shredded and ground in a tissue grinder (IKA A10, Germany) to fine powder. Ten grams of the powder were dissolved in 100 ml sterile double distilled water and heated for 1 h at 80 °C. The obtained extract was filtered through Rotilabo^®^ Typ 601P filter paper; the filtrate was collected in a 250 ml Erlenmeyer flask and then stored at 4 °C for further use (modified from (Verastegui et al., 1996).

### Green synthesis of silver and zinc oxide nanoparticles (Ag-NPs and ZnO-NPs)

Ag-NPs and ZnO-NPs were essentially synthesized as previously described (Babu and Prabu, 2011; Prakash et al., 2013; Sangeetha et al., 2011, 2012; Sun et al., 2014; Suzan et al., 2014; Vimala et al., 2014) using leaves (L) or fruits (F) extracts from *C. procera* resulting in the four different types of nanoparticles Ag-NPs-L, Ag-NPs-F, ZnO-NPs-L and ZnO-NPs-F. Solutions with silver nitrate or zinc nitrate (without *C. procera* extract) were also incubated at the same conditions and served as a negative control (Kumar et al., 2011).

To obtain Ag-NPs, 20 ml of a 1 mM AgNO_3_ (Sigma–Aldrich) solution were added drop-wise to 20 ml of the respective aqueous plant extract of *C. procera* under constant stirring at 80 °C within 30–45 min, for the reduction of Ag^+^ ions. This material was incubated in the dark (to minimize the photoactivation of silver nitrate) at 37 °C. The synthesis of ZnO-NPs was performed as previously described with some modifications. Briefly, 2 g of zinc nitrate (Sigma–Aldrich) was dissolved in 100 ml aqueous leaf or fruit extracts solution of *C. procera* under constant stirring. After complete dissolution of the mixture, the solution was kept under vigorous stirring at 80 °C for 2 h, subsequently allowed to cool at room temperature and the supernatant was discarded. Obtained NPs solutions were centrifuged at 4,500 rpm for 15 min after thorough washing and dried at 80 °C for 7–8 h. Crude pellets were then resuspended in sterile double distilled water, filtered through 0.2 μm filter and stored at 4 °C in the dark prior to their use.

### UV–vis spectrophotometry

The reduction of Ag^+^ ions or ZnO was assessed by measuring the UV–vis spectrum of 1 ml aliquots of sample in a cuvette as described earlier (Wiley et al., 2006). UV–vis spectral analysis for Ag-NPs and ZnO-NPs was carried out by measuring the optical density (OD) using the SPECTROstar^NANO^, BMG labtech, Germany scanning spectrophotometer. Measurements were performed between 220 and 1,000 nm with a resolution of 1 nm. Silver nitrate (1 mM) or zinc nitrate (2%) were used as a blank, respectively.

### Inductively coupled plasma mass spectrometry (ICP-MS)

Elements were determined in the samples after mineralization with nitric acid using ICP-MS. Briefly, the liquid samples (∼500 mg weighed to 0.1 mg) were placed in 12 ml quartz vessels, 1 ml subboiled nitric acid was added and the samples were placed in the autoclave (UltraCLAVE IV, EMLS, Leutkirch, Germany). Then the autoclave was pressurized with argon to 40 bars and the samples heated in 45 min to a temperature of 250 °C and kept at this temperature for 45 min. After cooling the samples were transferred into 15 ml polypropylene tubes (Greiner Bio-One). Zn (determined at *m*/*z* 66) and Ag (determined at *m*/*z* 107) were determined with ICP-MS (Agilent 7500ce, Agilent Technologies, Waldbronn, Germany) after appropriate dilution. The accuracy of the results was validated with the certified reference material 1640a (trace elements in water, NIST, Gaithersburg, ML, USA).

### Nanoparticle tracking analysis (NTA) measurements

To determine the size characteristics of the bio-synthesized nanoparticles NTA was performed using the NanoSight LM10 HS-488FT14 instrument (Malvern Instruments, Herrenberg, Germany). The nanoparticle solutions were diluted in filter-sterilized (0.02 mm) double distilled water and 300 μl of each sample was injected to the viewing unit using a disposable syringe. The concentration of the NPs was adjusted to a particle number between 10^6^ and 10^9^ particles per ml. A laser (wavelength 405 nm) illuminates the particles from aside, and the particles act as point scatters moving under Brownian motion. Despite rapid movements of the particles, they can be tracked by a conventional CCD camera. The recorded video can subsequently be analyzed analytically by a software program (NTA 2.3, Build 0025). The samples were measured for 60 s with manual shutter and gain adjustments. After capture of the diffusion coefficient and track lengths for the individual particles a quite accurate determination of the individual particle size can be made (ASTM, 2012; Patrick et al., 2013). Particle concentrations of the original nanoparticle solutions were calculated by the measured concentration of the diluted samples multiplied by the dilution factor.

### Determination of the minimum inhibitory concentration (MIC) by growth and INT reduction assay

Overnight cultures of *V. cholerae* or ETEC were subcultured 1:10,000 into LB. Samples of 100 μl bacterial culture were placed into 96-well plates and 10 μl of appropriate serial dilutions of Ag-NPs-L, Ag-NPs-F, ZnO-NPs-L or ZnO-NPs-F were added. At least two independent preparations of each NP type were tested. Leaf or fruit extracts alone were also tested and showed no effects compared to LB broth. An additional control consisted of NPs-free supernatants from NPs solutions obtained after two consecutive centrifugation steps (20,000 rpm, 4 h, 4 °C). The absence of NPs in the supernatant was confirmed by NTA. After 16 h incubation in a humid chamber at 37 °C, the optical density (OD_600_) was measured using the Spectrostar^NANO^ Microplate Reader (BMG Labtech). The MIC for growth was defined as the lowest concentration of NPs, which inhibited bacterial growth. Growth was defined by an at least 2-fold increase of the OD_600_ compared to the negative control (LB only). To confirm bacterial growth inhibition and determine lack of metabolic activity, 40 μL of p-iodonitrotetrazolium violet INT (0.2 mg/mL, Sigma–Aldrich) was added to microplate wells and reincubated at 37 °C for 30 min (Eloff, 1998). The MIC in the INT assay was defined as the lowest concentration of NPs that prevented color change as described earlier (Namrita et al., 2013).

### Growth kinetics and viability

Growth kinetics were essentially performed as previously described in transparent 24-well plates (Greiner) with 1 ml culture volume using LB broth, LB broth supplemented with leaf extract (10%) or LB broth supplemented with fruit extract (10%) starting at an OD_600_ = 0.01 (Moisi et al., 2013; Seper et al., 2011). These final concentrations of the plant extracts are at least 2-fold higher than the respective MIC of plant extracts with NPs. The OD_600_ was monitored every 30 min in the SPECTROstar^Nano^ microplate reader (BMG Labtech) at 37 °C with shaking. For presentation of data, at least six independent growth curves were monitored for each strain tested. The mean values were calculated and plotted. At 24 h viability in presence and absence of plant extracts was determined by plating appropriate dilutions of the cultures on LB plates. The obtained CFU/ml from at least three independent measurements are presented as mean ± standard deviation.

### Static biofilm assay

Static biofilms were performed in microtiter plates by crystal violet staining essentially as previously published (Seper et al., 2011), with some modifications. Briefly, the respective strains were grown over night on LB agar plates, suspended in LB, adjusted to an OD_600_ of 0.02. 130 μl of this dilution were placed in a 96 well microtiter plate (U bottom, Sterilin) for 24 h at RT. After 24 h, 20 μl of Ag-NPs-L, Ag-NPs-F, ZnO-NPs-L or ZnO-NPs-F solutions with concentrations of ∼ 10^8^ NPs/ml were added. Addition of 20 μl of LB broth, the plant leaf or fruit extracts from *C. procera* served as control. After another 24 h incubation at RT, wells were subsequently rinsed with dH_2_O using a microplate washer (Anthos Mikrosysteme GmbH, Fluido2), biofilm was stained with 0.1% crystal violet, solubilized in 96% ethanol and the OD_595_ was measured (SPECTROstar^NANO^, BMG Labtech) to quantify the amount of biofilm.

### Preparation of outer membrane proteins (OMPs) and whole-cell lysates (WCL)

OMPs were essentially prepared as previously published (Roier et al., 2013). Briefly, ON cultures of *V. cholerae* or ETEC grown in LB with or without sublethal concentrations of Ag-NPs-L or Ag-NPs-F (both 1 × 10^6^ NPs/ml) as well as ZnO-NPs-L or ZnO-NPs-F (both 1 × 10^5^ NPs/ml) respectively, as well as 0.025 mM of AgNO_3_ or 0.1 mM of Zn(NO_3_)_2_ solution were harvested by centrifugation (3,200 × *g*, 10 min, 4 °C), washed once in HEPES buffer (10 mM, pH 7.4) and resuspended in 1 ml HEPES buffer (10 mM, pH 7.4). Then the suspension was transferred in a cryo-tube and cells were disrupted by homogenization with 0.1 mm glass beads in combination with a PowerLyzer™ 24 (MO BIO Laboratories, Inc.), applying three times, 1 min cycles at 3400 rpm with 1 min intervals on ice between each cycle. Unbroken cells were removed by centrifugation (15,600 × *g*, 2 min, 4 °C). The supernatant containing the OMPs was transferred into a new tube and centrifuged again (15,600 × *g*, 30 min, 4 °C). The membrane pellet was re-suspended in 0.4 ml HEPES buffer (10 mM, pH 7.4). To solubilize the cytoplasmic membrane, 0.4 ml HEPES buffer (10 mM, pH 7.4) with 2% sarcosyl was added and incubated at room temperature (RT) for 30 min. After centrifugation (15,600 × *g*, 30 min, 4 °C), the pellet containing the OMPs was washed once with 0.5 ml HEPES buffer (10 mM, pH 7.4) and finally re-suspended in 50 μl HEPES buffer (10 mM, pH 7.4). Purified OMPs were stored at −20 °C.

The protein concentrations of OMP preparations were determined by photometric measurements of the absorbances at 260 nm and 280 nm using a Beckman Coulter DU730 spectrophotometer in combination with a TrayCell (Hellma) and the Warburg–Christian equation given as mg protein/ml = [(1.31 × A280) − (0.57 × A260)] × dilution factor (Warburg and Christian, 1941).

### SDS-PAGE and immunoblot analysis

To separate proteins the standard sodium dodecyl sulfate-polyacrylamide gel electrophoresis (SDS-PAGE) procedure in combination with 15% gels and the Prestained Protein Marker Broad Range (New England Biolabs) as a molecular mass standard was used (Laemmli, 1970). Approximately 5 μg protein was loaded for each sample. Proteins were stained according to Kang et al. (Kang et al., 2002) or transferred to a nitrocellulose membrane (Amersham) for immunoblot analysis, which was essentially performed as described previously (Roier et al., 2012), using anti-OmpU or anti-OmpT antisera generated in mouse (1:500 diluted in 10% skim milk) as primary and peroxidase-conjugated goat anti-mouse (diluted 1:10,000 in 10% skim milk, Dianova GmbH, Hamburg) as secondary antibody, respectively.

### Survival curve of *V. cholerae* and ETEC in the presence of Ag-NPs-L

For the time-dependent survival analysis, a bacterial overnight culture was diluted 1:10,000 using LB-Sm broth supplemented with aliquots of Ag-NPs-L (final concentration of 2.4 × 10^7^ or 1.2 × 10^7^ NPs/ml). Cultures were grown without agitation at 37 °C (using same conditions as for the MIC assay described above), and 100 μl were collected at the indicated time intervals, serially diluted in LB-Sm sterile broth and plated onto LB-Sm agar plates. Viable colonies were counted after 16 h at 37 °C. According to the volume plated on agar plates, the limit of detection for this assay was 10 CFU/mL.

### In vivo colonization assay

In vivo experiments were performed as previously described with some modifactions (Leitner et al., 2013; Moisi et al., 2009; Schild et al., 2007). CD-1 mice (Charles River Laboratories) were used in all experiments in accordance with the rules of the ethics committee at the University of Graz and the corresponding animal protocol, which has been approved by the Austrian Federal Ministry of Science and Research Ref. II/10b. Mice were housed with food and water ad libitum and monitored under the care of full-time staff. Mice were separated from their dams 1 h before infection. Subsequently, they were anesthetized by inhalation of isoflurane gas and then inoculated by oral gavage with 50 μl of *V. cholerae* or ETEC (approx. 1 × 10^5^ CFU/mouse for both pathogens). To determine the exact inputs appropriate dilutions of the inocula were plated on LB-Sm plates. 6 h post-infection, infected mice were divided into two groups. One group were treated orally with 50 μl of Ag-NPs–L (1.2 × 10^8^ NPs/ml), while the other group received 50 μl saline solution. After 24 h, the mice were sacrificed and the small intestine from each mouse was collected by dissection. The small intestine was mechanically homogenized in LB broth with 15% glycerol and appropriate 1:10 dilutions were plated on LB-Sm. After incubation at 37 °C ON, the colonization rates in CFU/small intestine were determined by back-calculation to the original volume of the homogenized small intestine.

### Statistical analysis

Data were analyzed using the Mann–Whitney *U* test or a Kruskal–Wallis test followed by post hoc Dunn's multiple comparisons. Differences were considered significant at P values of ≤0.05. For all statistical analyses, GraphPad Prism version 4.0a was used.

## Results

### Characterization of the nanoparticles

**Fig. 1:**
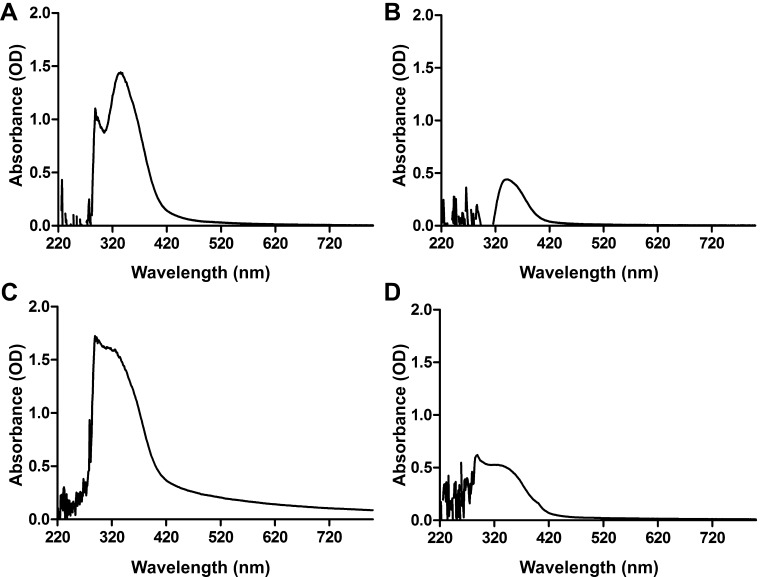
UV–vis spectra for the reaction mixture containing of nanoparticles (NPs) synthesized from *C. procera* leaves (L) and fruits (F). Shown are the UV–vis absorption spectra from 220 to 800 nm of ZnO-NPs-L(A), ZnO-NPs-F (B), Ag-NPs-L (C) and Ag-NPs-F (D).

**Fig. 2:**
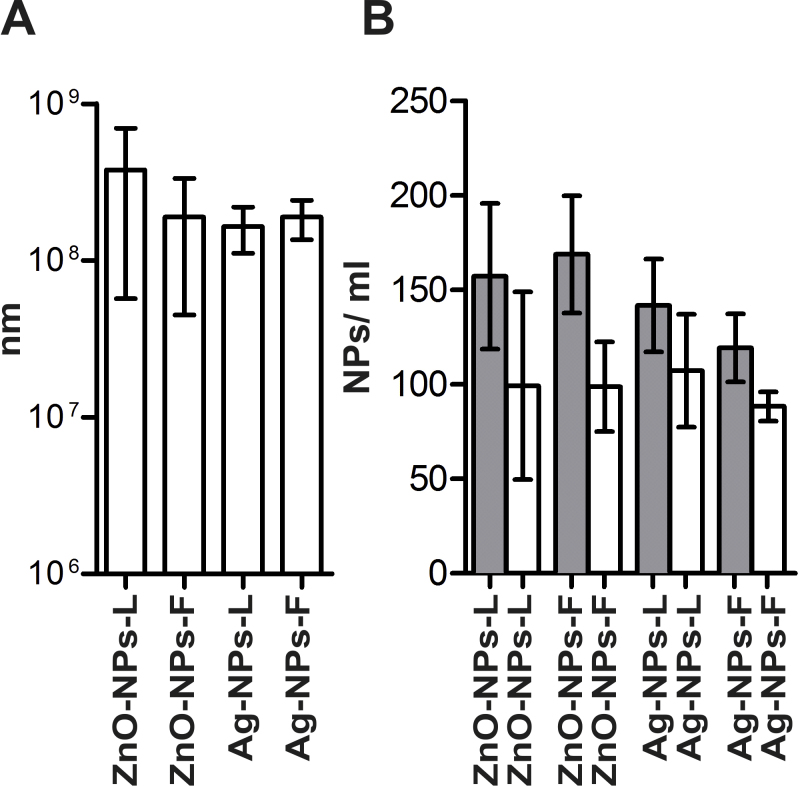
Characterization of biosynthesized zinc and silver nanoparticles by nanoparticles tracking analysis (NTA). Shown are the concentrations of NPs (A) as well as the NP sizes (B) presented as average (gray bars) and mode (white bars) NPs diameter sizes in nm. At least three independent preparations of each NP type were analyzed. The data is presented as the mean ± standard deviation (SD).

Zinc oxide and silver nanoparticles (ZnO-NPs and Ag-NPs) were synthesized according to established protocols using leaf (L) and fruit extracts (F) from *C. procera* (Geethalakshmi and Sarada, 2010; Hui et al., 2004; Sangeetha et al., 2011; Song and Kim, 2009), resulting in the four different types of nanoparticles ZnO-NPs-L, ZnO-NPs-F, Ag-NPs-L and Ag-NPs-F. After the addition of leaf and fruit extracts to the silver or zinc nitrate solutions, color changes appeared within 30 min indicating the completion of the reaction, which is due to the excitation of plasmon vibrations in the metal nanoparticles (data not shown). In contrast, the control silver or zinc nitrate solution without extracts showed no color change (data not shown). The intensity of colors steadily increased along the incubation period. Finally, Ag-NPs-L and Ag-NPs-F solutions exhibited a dark brown color, while solutions of Zn-NPs-L and Zn-NPs-F exhibited dark yellow color. This may be due to the excitation of the surface plasmon resonance (SPR) effect (Haes and Van Duyne, 2002) and the reduction of either AgNO_3_ (Mulvaney et al., 1996) or zinc nitrate (Sangeetha et al., 2011). The reduction of aqueous extracts by silver or zinc ions and the formation of each NP-type were confirmed using UV–vis spectroscopy (Fig. 1). A wavelength scans in the UV–vis spectra revealed an absorption peak at approximately *λ* = 340 for ZnO-NPs-L and Zn-NPs-F (Fig. 1A and B). Furthermore Ag-NPs-L and Ag-NPs-F exhibited characteristic absorption peaks at approximately *λ* = 370 nm as previously published (Jeeva et al., 2014) (Fig. 1C and D). The presence of Zn and Ag in the NPs solutions was confirmed by inductively coupled plasma mass spectrometry (ICP-MS), which revealed an at least 7-fold increase in case of Zn or 400-fold in case of Ag in the NPs solutions compared to the plant extracts, respectively (Table 1). The exact size distributions and concentrations of independent NP preparations used in the assays presented herein were determined by nanoparticle tracking analysis (Fig. 2). Throughout the study, each type of NP has been prepared at least three times without tremendous changes in yield or quality, suggesting a reproducible production of the NPs. In general, all NP preparations showed similar results with mean concentrations ranging from 1.65 to 3.8 × 10^8^ NPs/ml, mode particle sizes of 88–100 nm and average particle size of 120–169 nm. The difference between mode and average particle sizes indicates a non-parametric distribution of the NPs with the majority ranging around 90–100 nm in size as well as a minor population with bigger diameters. No aggregations or debris were detected by visualization of the NPs within the nanoparticle tracking analysis (data not shown), which indicates that NP suspensions are quite pure and homogenous.

### Antimicrobial activity of ZnO-NPs and Ag-NPs against *V. cholerae* and ETEC.

**Fig. 3:**
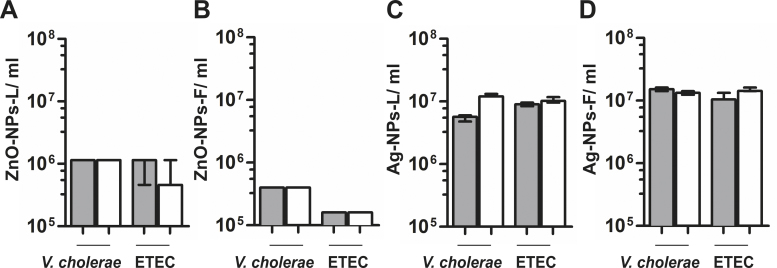
Antibacterial activity of ZnO-Nps and Ag-Nps against *V. cholerae* and ETEC. Minimal inhibitory concentrations (MIC) for growth (gray bars) and metabolic activity (open bars) against *V. cholerae* and ETEC were determined for ZnO-NPs-L (A), ZnO-NPs-F (B), Ag-NPs-L (C) and Ag-NPs-F (D). MIC for growth was determined by measuring OD_600_ measurement and MIC for metabolic activity was assessed by a tetrazolium reduction assay. Please refer to the materials and methods section for details. Shown are the medians from at least eight independent measurements. The error bars indicate the interquartile range.

**Fig. 4:**
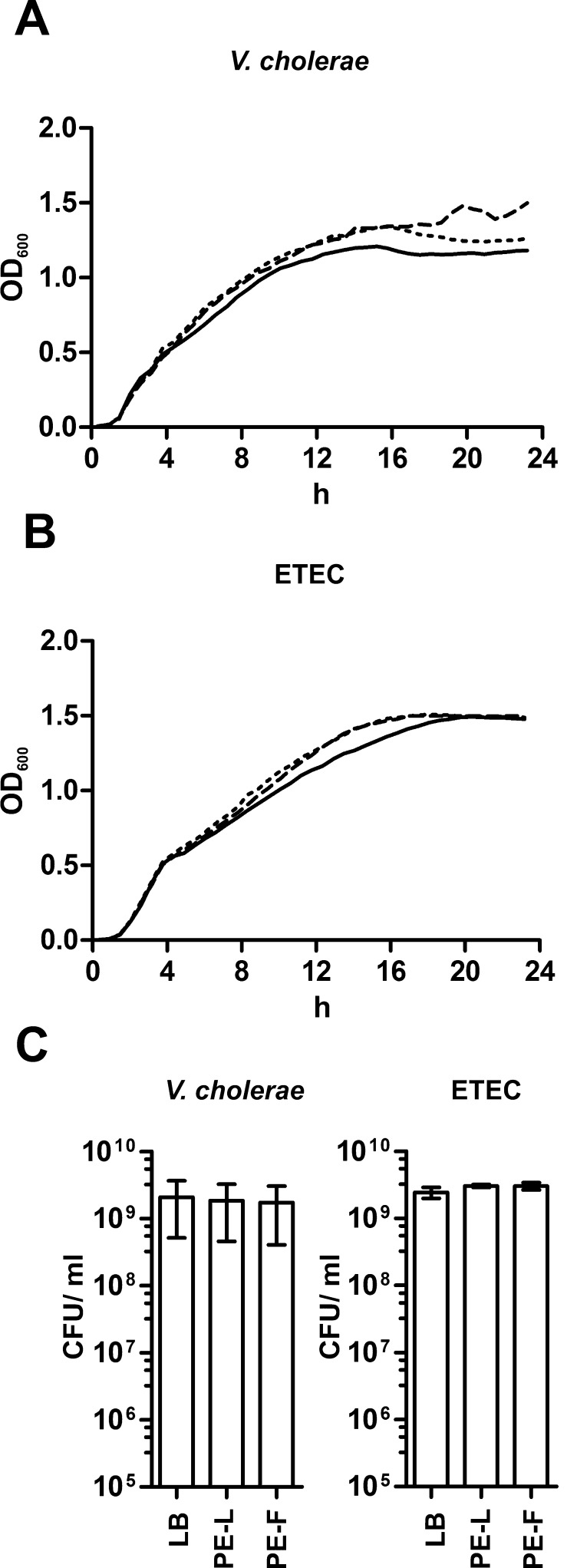
*C. procera* plant extracts do not affect growth or viability of *V. cholerae* and ETEC. Shown are the growth kinetics of *V. cholerae* (A) and ETEC (B) in LB broth with leaf (dashed line) or fruit extracts (dotted line). LB broth alone served as control condition (solid line). Shown are the medians from at least six independent measurements. (C) At 24 h the CFU/ml was determined by plating appropriate dilutions on LB plates. Data represent mean ± standard deviation (SD) of at least three independent measurements.

Antibacterial activity of the different NPs synthesized by *C. procera* plant extracts against the human pathogens *V. cholerae* and ETEC was analyzed by minimal inhibitory concentration (MIC) and INT assays (Fig. 3). MIC was defined as the lowest concentration at which no increase of the OD_600_ of the pathogens was observed. However, in the absence of cell lysis the measurement of turbidity cannot distinguish between live and dead bacteria (Carroll et al., 2010). Thus, we additionally determined bacterial viability by a colorimetric INT-formazan assay, which allows detection of viable bacteria by their respiratory activity (Mann and Markham, 1998; Palomino et al., 2002). All four NPs showed reproducible, effective antibacterial activity with similar results in both assays (Fig. 3). In general, ZnO-NPs showed a slightly higher efficacy compared to Ag-NPs, with ZnO-NPs-F exhibiting the lowest MIC against both pathogens. In detail, ZnO-NPs concentrations of 1.6 × 10^5^–1.2 × 10^6^ per ml were sufficient for killing of *V. cholerae* and ETEC (Fig. 3A and B), while in case of the Ag-NPs concentration of 5 × 10^6^–1.2 × 10^7^ per ml were necessary (Fig. 3C and D). Within the MIC and INT assays no detectable difference compared to the LB control were observed in the presence of plant extracts alone (data not shown). Consistently, a comprehensive analysis revealed that neither the presence of leaf nor fruit extracts had a negative effect on the growth kinetics or viability of *V. cholerae* or ETEC in comparison to the LB control (Fig. 4). To exclude the possibility of soluble side-products with antimicrobial activity in the NPs solution, the NPs-free supernatants from NPs solutions were obtained by centrifugation and tested in the MIC/INT assay. The absence of NPs in the supernatant was confirmed by NTA. None of the NP-free supernatants derived from the four types of NPs solutions (ZnO-NPs-L, ZnO-NPs-F, Ag-NPs-L and Ag-NPs-F) exhibited any residual antimicrobial activity. Thus, the determined MIC correlates with the presence of NPs.

### ZnO-NPs and Ag-NPs have inverse effects on biofilm formation

**Fig. 5:**
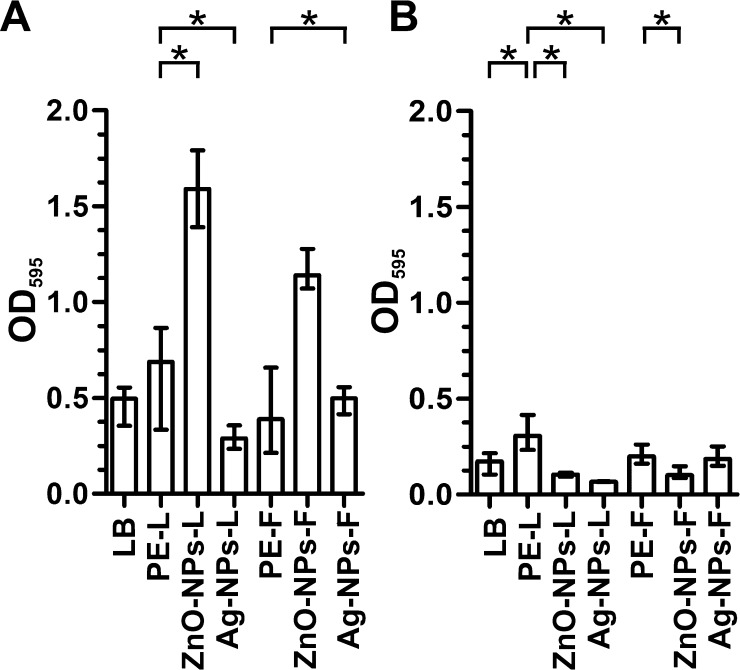
Impact of NPs on biofilms of *V. cholerae* and ETEC. *V. cholerae* (A) and ETEC (B) biofilms were allowed to grow for 24 h before ZnO-NPs-L, ZnO-NPs-F, Ag-NPs-L or Ag-NPs-F were added. Addition of LB broth (LB), plant extracts from leaves (PE-L) or fruits (PE-F) served as a control. After an additional 24 h biofilm formation was quantified by crystal violet staining and subsequent determination of the OD_595_. Shown are the medians from at least eight independent measurements. The error bars indicate the interquartile range. Significant differences between the data sets are marked by asterisks (*P* < 0.05; Kruskal–Wallis test and post hoc Dunn's multiple comparisons).

Biofilm formation is an important survival strategy and persistence mode between epidemic outbreaks of the facultative human pathogens *V. cholerae* and ETEC (Ahmed et al., 2013; Colwell et al., 2003). Recent findings suggest, that biofilms are a likely form in which *V. cholerae* and ETEC are taken up by humans, providing a concentrated infective dose (Ahmed et al., 2013; Colwell et al., 2003; Hall-Stoodley and Stoodley, 2005; Huo et al., 1996; Seper et al., 2011; Tamayo et al., 2010). Since biofilms are generally known to promote resistance against several antimicrobial agents (Lewis, 2005, 2008), we analyzed the impact of NPs on *V. cholerae* or ETEC biofilms. Therefore, we allowed *V. cholerae* or ETEC to form static biofilms for 24 h before ZnO-NPs-L, ZnO-NPs-F, Ag-NPs-L or Ag-NPs-F were added. Addition of LB broth, *C. procera* fruit or leaf extract served as controls, respectively. Finally, after an additional incubation period of 24 h the biofilm amount was quantified by crystal violet staining (Fig. 5). Addition of plant extract (fruit or leaf) had either no or a beneficial effect on biofilm formation in comparison to the LB control. Thus, the NPs treated biofilms were compared with the appropriate fruit or leaf plant extract, respectively. Addition of Ag-NPs-F showed no change in biofilm formation of both pathogens (Fig. 5A and B). In case of ETEC, treatment with ZnO-NPs-L, ZnO-NPs-F and Ag-NPs-L significantly reduced the biofilm amount compared to the plant extract treated control, respectively (Fig. 5B). This could also be observed for Ag-NPs-L treated biofilms of *V. cholerae* (Fig. 5A). In contrast, treatment with ZnO-NPs-L and ZnO-NPs-F had an opposite effect and significantly increased the biofilm amount of *V. cholerae*.

**Fig. 6:**
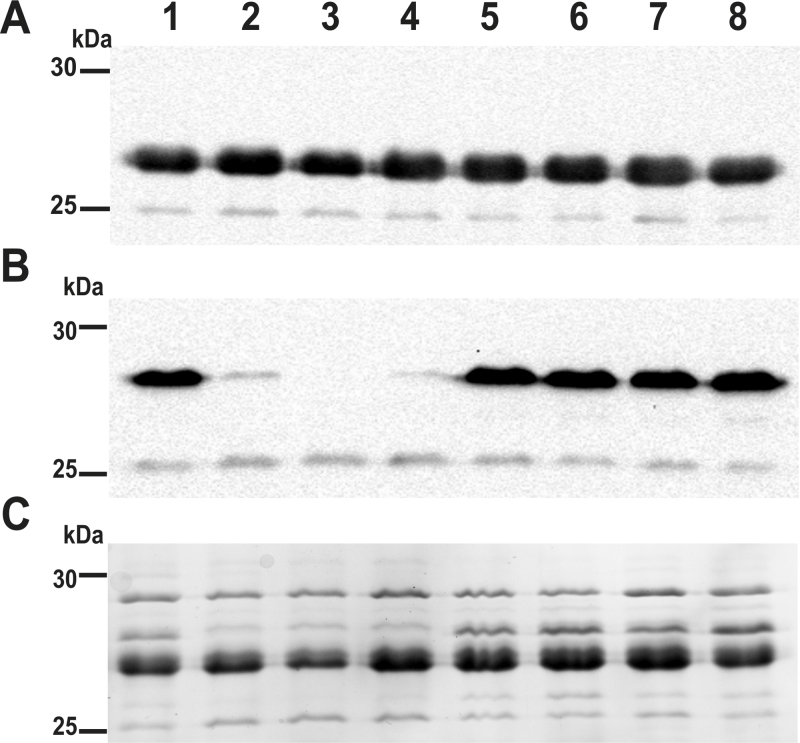
Immunoblot analysis of outer membrane preparations derived from *V. cholerae* treated with NPs. Depicted are the immunoblots for the detection of OmpU (A) or OmpT (B) as well as the protein profiles after Kang-staining (C) of OM preparations from *V. cholerae* grown in LB broth without supplements (lane 1) or supplemented with zinc nitrate (lane 2), ZnO-NPs-L (lane 3), ZnO-NPs-F (lane 4), Ag-NPs-L (lane 5), Ag-NPs-F (lane 6), *C. procera* leaf extract (lane 7) or *C. procera* fruit extract (lane 8). Samples (approx. 6 μg protein each) were separated by SDS-PAGE (15% gels) and protein bands were visualized according to Kang et al. (2002). Lines to the left indicate the molecular masses of the protein standards in kDa.

The pronounced increase in biofilm formation of *V. cholerae* treated with ZnO-NPs was unexpected. However, it was recently shown that adenylyl cyclases can be inhibited by Zn and consequently a sublethal concentration of ZnO-NPs could decrease cAMP levels in *V. cholerae* (Klein et al., 2002, 2004). Besides others, the second messenger cAMP negatively effects biofilm formation as well as expression of the cholera toxin and the major colonization factor TCP in *V. cholerae* (Fong and Yildiz, 2008; Skorupski and Taylor, 1997a, 1997b). Thus, treatment with sublethal concentrations of ZnO-NPs could cause adverse effects and induce biofilm formation and virulence. Interestingly, the outer membrane porin OmpT is positively regulated by this second messenger and lack of cAMP completely abolishes OmpT expression (Li et al., 2002). In order to confirm the hypothesis of adenylyl cyclase inhibition by ZnO-NPs, the outer membrane (OM) proteins were isolated from *V. cholerae* cultures grown in absence or presence of sublethal concentrations of ZnO-NPs, Ag-NPs or zinc- and silver nitrate solutions. Subsequently, these OM preparations were subjected to immunoblot analysis (Fig. 6). Kang staining and detection of OmpU by immunoblot served as loading controls (Fig. 6A and C). While no difference in the abundance of OmpU was observed, all preparations grown in presence of ZnO-NPs or zinc nitrate exhibited only low levels of OmpT (Fig. 6B, lane 2, 3, 4). This result strengthens the hypothesis that treatment with ZnO-NPs inhibits the adenylyl cyclase activity resulting in low cAMP levels.

### Ag-NPs-L kills *V. cholerae* faster than ETEC

**Fig. 7:**
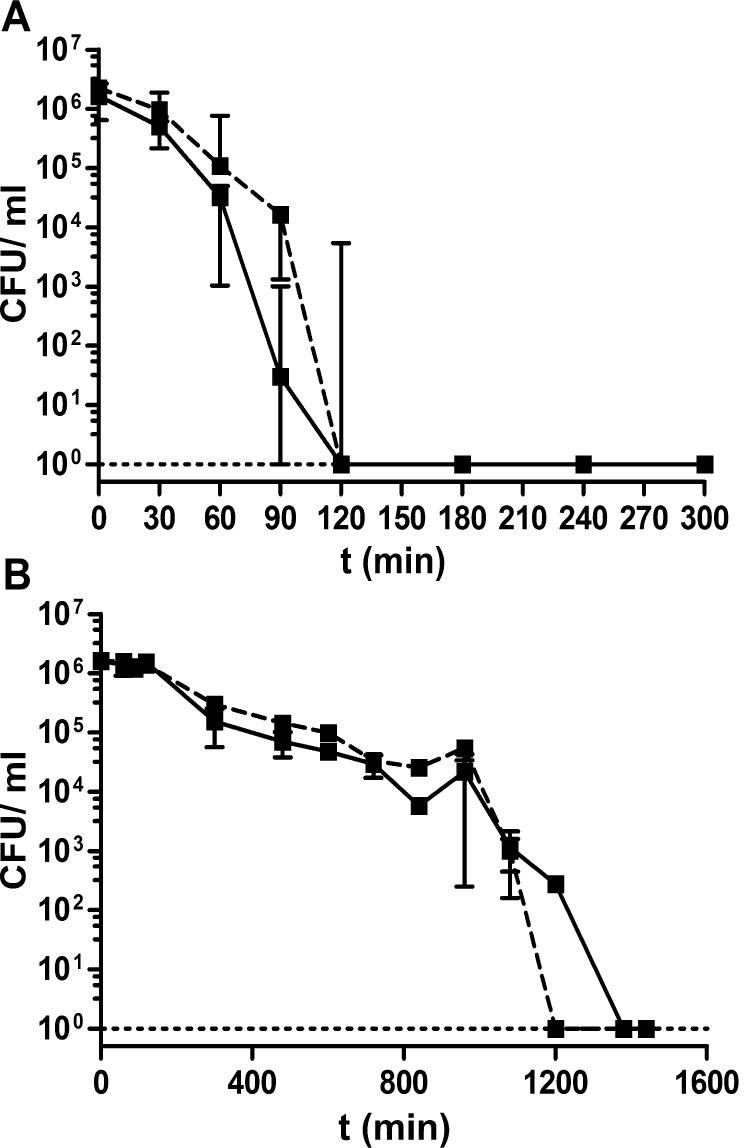
Survival curve of *V. cholerae* and ETEC in presence of Ag-NPs-L. Shown are the median CFU of *V. cholerae* (A) and ETEC (B) in LB broth supplemented with 2.4 × 10^7^ Ag-NPs-L/ml (solid line) or 1.2 × 10^7^ Ag-NPs-L/ml (dashed line) over time. Shown are the medians from at least three independent measurements. The error bars indicate the interquartile range of each data set for each time point. The limit of detection for this assay was 10 CFU.

Based on these results ZnO-NPs may interfere in cAMP signaling and cause adverse effects. In addition, Ag-NPs-F had no impact on biofilm formation of both pathogens. Thus, Ag-NPs-L were selected for further analysis. First, the killing dynamic of Ag-NPs-L against log-phase cultures of *V. cholerae* and ETEC was determined (Fig. 7). Two different concentrations of Ag-NPs-L were tested, with one close to the MIC and the other 2-fold higher. Addition of Ag-NPs-L at these two different concentrations to a *V. cholerae* culture resulted in both cases in a rapid, continuous drop of viable cells with no detectable CFUs after 2 h (Fig. 7A). In the case of ETEC, killing was delayed with a steady decrease of CFUs over time for both concentrations tested. Finally no detection of viable cells could be observed after 20 h for the higher or 22 h for the lower concentration of Ag-NPs-L, respectively (Fig. 7B). Based on these results, Ag-NPs-L kill *V. cholerae* faster than ETEC. Furthermore, concentrations above the MIC do not shorten the duration of killing.

### Oral treatment with Ag-NP_S_-L reduces colonization of *V. cholerae* and ETEC.

**Fig. 8:**
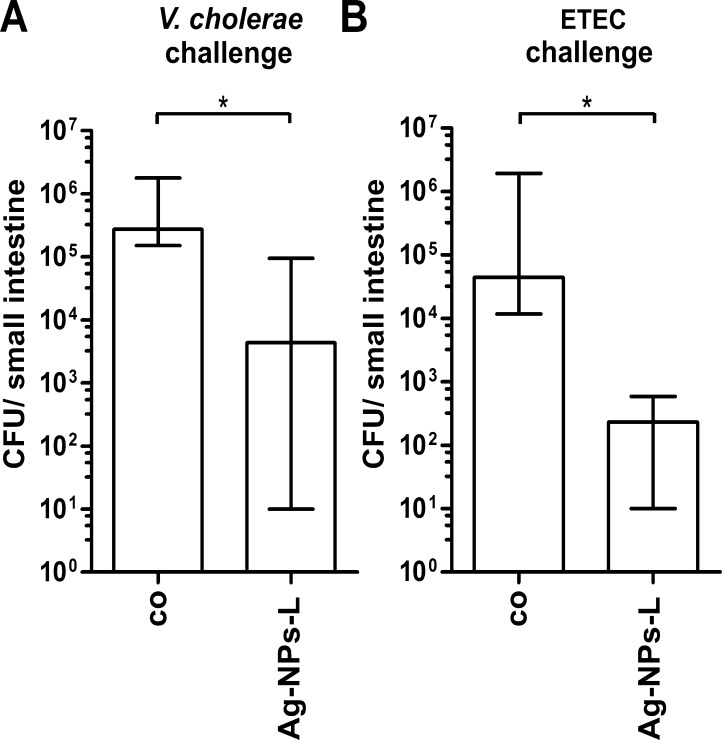
Impact of Ag-NPs-L on colonization fitness of *V. cholerae* and ETEC. Shown are the recovered CFU per small intestine from mice orally infected with either *V. cholerae* (A) or ETEC (B). 6 day old CD-1 mice were inoculated with either *V. cholerae* or ETEC (approx. 1.5 × 10^6^ CFU per mouse for both pathogens). At 6 h post infection the control group (co) received 50 μl saline while treatment group received 50 μl Ag-NPs-L solution (1.2 × 10^8^ NPs/ml). At 24 h post infection, the small intestines were collected, homogenized in LB medium and plated for CFU counting. Each column represents the recovered CFU per small intestine from at least eight mice. The horizontal bars indicate the median of each data set. Significant differences between the data sets are marked by asterisks (*P* < 0.05; Kruskal–Wallis test and post hoc Dunn's multiple comparisons).

Finally, we addressed the impact of Ag-NPs-L on the small intestine colonization of *V. cholerae* or ETEC using the infant mouse model. Therefore, mice were infected with *V. cholerae* or ETEC and orally treated with an Ag-NPs-L solution 6 h post-infection. The control group received an equal volume of saline buffer, which was also administered orally. The number of *V. cholerae* CFU recovered from the small bowel at 24 h post-infection are shown in Fig. 8. All control mice were stable colonized with median colonization levels of 3 × 10^5^ for *V. cholerae* or 5 × 10^4^ CFU for ETEC, respectively. Treatment with Ag-NPs-L resulted in a significant decrease in the colonization levels, with a 50-fold reduction in case of *V. cholerae* and 200-fold reduction for ETEC compared to the control groups.

## Discussion

Several approaches have been employed to improve the methods for synthesizing Ag- and ZnO-NPs including chemical and biological methods. Recently, nanoparticles synthesis based on plant extracts is becoming more popular (Abasaheb et al., 2013; Suzan et al., 2014; Vimala et al., 2014). For example, Ag-NPs were prepared from *Aloe vera* extract after 24 h of incubation or from *Acalypha indica* leaf extract after only 30 min of incubation using aqueous solutions of silver nitrate (Chandran et al., 2006; Krishnaraj et al., 2010). In the current study, Ag- and ZnO-NPs were rapidly synthesized from leaf and fruit extracts of *C. procera* within 2 h of incubation. The main mechanism is based on a plant-assisted reduction due to *C. procera* phytochemicals including flavones, organic acids, and quinones that are water soluble and are responsible for the immediate reduction of the ions (Iravani, 2011; Jha et al., 2009; Prabhu and Poulose, 2012). The formation of polydispersed nanoparticles with concentrations of approx. 10^8^ NPs/ml in the aqueous solution was confirmed by UV–vis spectrometry and NTA analysis. The obtained yield, particle size and general quality is consistent with recent reports using aqueous extracts of neem and triphala leaves for synthesis of Ag-NPs or ZnO-NPs (Chandran et al., 2006; Ponarulselvam et al., 2012) (Asmita et al., 2012; Prabhu and Poulose, 2012; Sangeetha et al., 2011; Suzan et al., 2014; Vimala et al., 2014). It was also concluded that nanoparticle solutions were stable for more than six months with little signs of aggregation (Ankanna et al., 2010; Ponarulselvam et al., 2012; Saifuddin et al., 2009).

The antibacterial activities of phyto-synthesized Ag-NPs and ZnO-NPs were studied against the two Gram-negative pathogens *V. cholerae* and ETEC, which are dominant bacterial causative agents for diarrheal diseases. Although both NPs exhibited robust antibacterial activity in the MIC and INT assays, the ZnO-NPs generally showed a slightly higher efficacy against both pathogens compared to Ag-NPs. Interestingly, the amount of NPs necessary for growth inhibition (MIC assay) and inhibition of metabolic activity (INT assay) was almost equal, suggesting that the dominant antimicrobial target of the NPs are metabolic pathways of the bacteria. Noteworthy, antibiotic resistance of bacterial biofilms may be caused by poor antibiotic penetration within the biofilm matrix, an altered microenvironment or an adaptive bacterial response. Such mechanisms acting together can raise the antibiotic resistance of biofilms by up to 1000 times in comparison with free living bacterial cells (Mah and O’Toole, 2001). The aforementioned attributes of biofilms place them amongst the most serious problems, which medicine is currently facing. Thus, considerable effort has been made to identify novel technologies that could form the basis of anti-biofilm therapies, which are superior to current antibiotic treatment strategies. As a potential application for water disinfection the NPs should also affect biofilms of these pathogens.

Interestingly, addition of ZnO-NPs to *V. cholerae* enhanced biofilm formation. A possible explanation might be the inhibition of the adenylyl cyclase by Zn-based compounds (Klein et al., 2002, 2004). Consequently, cAMP levels are decreased, which de-represses biofilm formation in *V. cholerae*. Consistent with this hypothesis we found only low levels of the cAMP-induced porin OmpT in the OM of *V. cholerae* treated with sublethal concentrations of ZnO-NPs. Noteworthy, the second messenger cAMP is involved in multiple regulatory pathways in *V. cholerae* including natural competence, chitin colonization, outer membrane composition, motility, biofilm formation and virulence gene expression (Antonova et al., 2012; Blokesch, 2012; Liang et al., 2007; Nielsen et al., 2010). Although in most cases the exact mechanisms are not yet fully understood, it was reported earlier that cAMP negatively affects biofilm formation as well as expression of the cholera toxin and the major colonization factor TCP (Fong and Yildiz, 2008; Skorupski and Taylor, 1997a, 1997b). Since, ZnO-NPs treatment might cause adverse effects by unleashing biofilm formation or virulence; we did not pursue other studies on ZnO-NPs.

In contrast to ZnO-NPs, treatment with Ag-NPs-L significantly reduced biofilms of ETEC and *V. cholerae*. Consistent with our results, Ag-NPs have been recently shown to inhibit and reduce biofilm formations of several bacterial species (Markowska et al., 2013). For example, studies in rabbits showed that nanoparticle silver ion-coated implants inhibited *Staphylococcus aureus* biofilm formation without causing silver accumulation in host tissues, even 28 d after impregnation (Gupta et al., 2014; Kalishwaralal et al., 2010; Markowska et al., 2013; Secinti et al., 2011).

Besides ex vivo application for biofilm reduction and water disinfection, we also demonstrated that oral administration of Ag-NPs-L can lower the colonization levels of *V. cholerae* and ETEC in vivo. Gastrointestinal intake of NPs may indeed occur through nano-technological food, packaging or medical applications. Still, inhalation accounts for the majority of NPs exposure routes in humans, as NPs can be released into air in occupational settings (Klien and Godnic-Cvar, 2012). Consistent with other reports, we observed no adverse side effects by single oral administration of Ag-NPs, which suggests that the colloidal Ag-NPs-L had no acute toxic effects (Quang et al., 2013). For example, Pattwat and coworkers reported that the colloidal Ag-NPs was found to be nontoxic when oral, ocular and dermal toxicity tests in mice and guinea pigs were performed (Pattwat et al., 2011). The repeated-dose toxicity was assayed in rats and mice given Ag-NPs for 28-days (Eun-Jung et al., 2010; Kim et al., 2008). While no significant changes in body weight could be observed, exposure to high doses of more than Ag-NPs (300 mg/kg for rats and 1 mg/kg in mice) resulted in adverse effects indicating slight liver or kidney damages. In general, the accumulation in tissues seems to occur in a dose-dependent manner only after repeated treatment (Quang et al., 2013).

This study provides a first characterization of the potential applications of nanoparticles as antibacterial therapeutic agents against *V. cholerae* and ETEC. Based on our results Ag-NPs offer an inexpensive alternative approach to reduce the infectious dose of the pathogens in contaminated water and might also be applicable for in vivo therapy of diarrheal diseases. However, further *in vitro* and molecular studies are needed to elucidate the clear evidence of toxic mechanisms that will be related to Ag^+^. Further long-term toxicity, mutagenicity and carcinogenicity studies are required to clarify any adverse effects and are necessary to support the safe use of colloidal Ag-NPs. The fast and simple synthesis of nanoparticles does not require special trained staff or expensive equipment and is likely to be performed in epidemic areas. In contrast to antibiotics, prolonged exposure of bacteria to silver nanoparticles has not resulted in the development of resistant cells so far. Silver nanoparticles as biocides tend to target multiple sites on or within bacterial cells and hence have a broad spectrum activity (Markowska et al., 2013). Thus, the application of silver nanoparticles as an effective antimicrobial agent should not cause microbial resistance even after long-term usage.

## Figures and Tables

**Table 1:** Analysis of plant extracts as well as biosynthesized zinc and silver nanoparticles by inductively coupled plasma mass spectrometry (ICP-MS).

Plant extracts/nanoparticles	Total concentration Zn (mg/kg) mean ± SD^a^	Total concentration Ag (mg/kg) mean ± SD
Leaf extract	1.4 ± 0.1	<0.005
Fruit extract	0.98 ± 0.14	<0.005
Zn-NPs-L	9.24 ± 0.54	n.d.^b^
Zn-NPs-F	25.6 ± 0.8	n.d.
Ag-NPs-L	n.d.	2.1 ± 0.8
Ag-NPs-F	n.d.	10 ± 3

a Results are given as mean ± standard deviation of at least three independent preparations.

b Not determined.
